# Effects of the *PCSK9* C378W Mutation on PCSK9 Levels and Lipid Profiles in Taiwanese Individuals: A Loss-of-Function Mutation with Potential Cardiovascular Benefits

**DOI:** 10.3390/genes16091113

**Published:** 2025-09-19

**Authors:** Semon Wu, Lung-An Hsu, Kuan-Hung Yeh, Yu-Lin Ko

**Affiliations:** 1 Department of Life Science, Chinese Culture University, Taipei 11114, Taiwan; wsm4@ulive.pccu.edu.tw; 2 The First Cardiovascular Division, Department of Internal Medicine, Chang Gung Memorial Hospital, Taoyuan 33305, Taiwan; hsula@cgmh.org.tw; 3 College of Medicine, Chang Gung University, Taoyuan 33305, Taiwan; 4 The Division of Cardiology, Department of Internal Medicine, Taipei Tzu Chi Hospital, Buddhist Tzu Chi Medical Foundation, New Taipei City 23142, Taiwan; yeh2644@tzuchi.com.tw; 5 School of Medicine, Tzu Chi University, Hualien 97004, Taiwan; 6 Department of Research, Taipei Tzu Chi Hospital, Buddhist Tzu Chi Medical Foundation, New Taipei City 23142, Taiwan

**Keywords:** *PCSK9*, low-density lipoprotein cholesterol level, loss-of-function mutation, PCSK9 level, C378W mutation

## Abstract

**Background**: Proprotein convertase subtilisin/kexin type 9 (PCSK9) is a key regulator of lipid metabolism. The rare PCSK9 C378W (rs776752113) mutation influences the level of low-density lipoprotein cholesterol (LDL-C); however, its association with PCSK9 levels remain unclear. **Methods**: This study investigates the frequency of the C378W mutation and its effects on PCSK9 levels and lipid profiles in 5901 Taiwan Biobank participants, including 1486 individuals with available whole-genome sequencing data. The C378W mutations were detected using a TaqMan genotyping assay and confirmed using direct DNA sequencing. **Results**: Whole-genome sequencing data revealed a single carrier of the C378W mutation. The TaqMan assay identified seven carriers of the 378W allele (7/5901 [0.119%]). After the exclusion of an individual with a history of hyperlipidemia, six carriers exhibited significantly lower levels of LDL-C (−30.5%) and PCSK9 (−56.4%) than noncarriers (LDL-C: 81.17 ± 21.79 vs. 116.70 ± 30.70 mg/dL [*p* = 0.0005]; PCSK9: 67.20 ± 14.83 vs. 154.02 ± 45.52 ng/mL [*p* = 3.59 × 10^−12^]. Moreover, carriers exhibited significantly lower levels of total cholesterol (−18.6%) and non-high-density lipoprotein cholesterol (non-HDL-C; −28.4%) than noncarriers (total cholesterol: 157.17 ± 19.30 vs. 193.18 ± 35.22 mg/dL [*p* = 0.0035]; non-HDL-C: 99.50 ± 20.22 vs. 138.91 ± 34.97 mg/dL [*p* = 0.0005]). Mediation analysis suggests that the association between the C378W mutation and LDL-C levels persisted even after adjustment for PCSK9 levels. Functional characterization indicates that the C378W mutation impairs protein stability and function. **Conclusion**: In conclusion, the rare C378W mutation represents a loss-of-function mutation in the Taiwanese population. This variant is independently associated with reduced PCSK9 levels and improved lipid profiles, highlighting its potential cardioprotective role.

## 1. Introduction

Proprotein convertase subtilisin/kexin type 9 (PCSK9), a multifaceted serine protease predominantly synthesized by the liver, plays a pivotal role in lipid metabolism by regulating the degradation and recirculation of low-density lipoprotein (LDL) receptor [1,2]. Beyond its systemic effects on lipid profiles, PCSK9 facilitates atherosclerotic plaque development and thrombosis by recruiting leukocytes and activating coagulation pathways [2,3]. Circulating PCSK9 levels exhibit ~100-fold variation across individuals [4], with nongenetic factors accounting for 23% of the total variance [4], and heritability estimates ranging from 22% to 47% [5,6,7]. Statins markedly upregulate PCSK9 expression [8,9,10]. Notably, epigenetic silencing has been demonstrated to reduce PCSK9 levels in experimental models [11]. In addition to the *PCSK9* gene, multiple novel candidate loci that influence PCSK9 levels have been identified in genome-wide association studies and meta-analyses [6,12].

Human *PCSK9* is located on chromosome 1p32.3 [13]. More than 850 *PCSK9* mutations have been reported so far, with loss-of-function mutations and disease-related dominant gain-of-function mutations exerting opposing effects on the level of LDL cholesterol (LDL-C) and the risk of coronary heart disease [2,14,15]. Ethnic disparities in *PCSK9* variants are commonly observed between Caucasian and East Asian populations [16,17,18,19,20,21,22,23,24,25]. Despite the established role of PCSK9, the effects of its rare genetic variants on PCSK9 function, lipid profiles, and cardiovascular outcomes remain unclear.

Recently, a rare *PCSK9* mutation resulting from a heterozygous C-to-G substitution at the 1134th base of PCSK9 has been reported in individuals of Uyghur and Han Chinese descent; this mutation results in the substitution of Cys at the C378 position with Trp (PCSK9:NM_174936:exon7:c.1134C>G:p.C378W; rs776752113) and is associated with reduced levels of LDL-C [26]. The present study investigates the frequency and functional implications of the C378W mutation and its effects on PCSK9 levels and lipid profiles in the Taiwanese population.

## 2. Materials and Methods

### 2.1. Study Cohort

**Figure 1:**
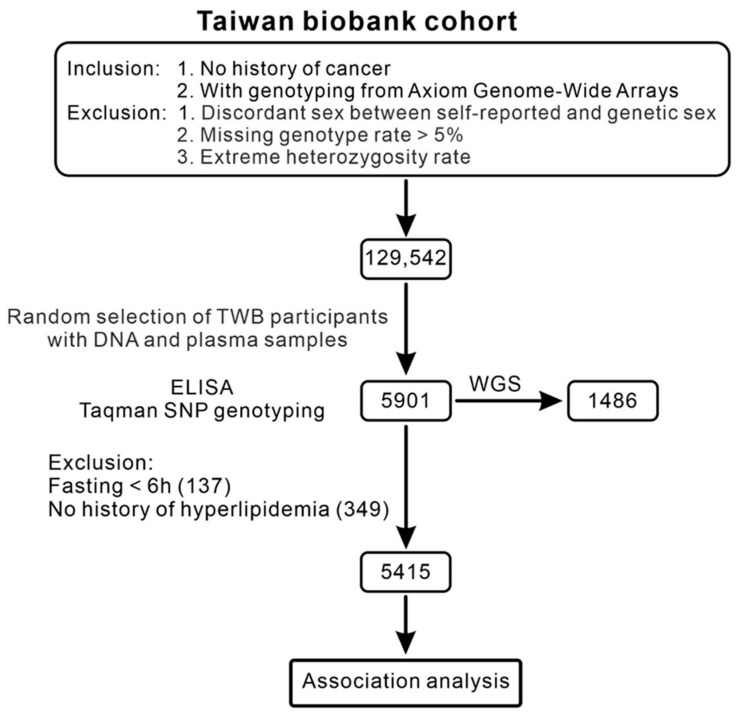
Flowchart depicting participant selection.

Relevant data were collected from 129,542 Taiwan Biobank (TWB) participants who did not have cancer. Eligible participants were recruited across Taiwan from 2008 to 2015. All participants provided written informed consent before participation. A random subset of 5901 TWB participants who had both DNA and plasma samples available were included in this study to determine the allele frequency of the *PCSK9* C378W (rs776752113) variant and evaluate the level of PCSK9. Within this subset, 1486 participants had whole-genome sequencing (WGS) data available. Participants with a history of hyperlipidemia or a fasting duration of <6 h were further excluded from the analyses of the associations between the rs776752113 variant, lipid profiles, and PCSK9 levels. A flowchart depicting the enrollment process is presented in Figure 1. The study protocol was approved by the Research Ethics Committee of Taipei Tzu Chi Hospital (approval numbers: 08-XD-005) and the Ethics and Governance Council of the TWB (approval numbers: TWBR11107-03). This study adhered to the ethical principles outlined in the Declaration of Helsinki.

Among 129,542 TWB participants, 5901 with available DNA and plasma samples were randomly selected for SNP genotyping and ELISA, of whom 1486 also had whole-genome sequencing (WGS) data. After excluding individuals with fasting duration <6 h or a history of hyperlipidemia, 5415 participants were included in the association analysis.

### 2.2. Lipid Profiles and PCSK9 Levels

Characteristics such as age, body mass index (BMI), sex, and smoking status were documented at baseline. Lipid profiles such as the levels of total cholesterol (TC), high-density lipoprotein cholesterol (HDL-C), and triglycerides (TG) were measured through colorimetric assays (Hitachi LST008; Automatic Clinical Chemistry Analyzer; Hitachi, Naka, Japan). The level of low-density lipoprotein cholesterol (LDL-C) was measured as follows: If the TG level was ≤350 mg/dL, the level of LDL-C was determined by subtracting the sum of HDL-C and one-fifth of TG levels from TC. If the TG level was >350 mg/dL, direct measurements of LDL-C levels were conducted using colorimetric assays. The level of non-HDL-C was calculated by subtracting the level of HDL-C from that of TC, and the level of remnant cholesterol was calculated by subtracting the level of LDL-C from that of TC. Plasma levels of PCSK9 were measured using a solid-phase sandwich enzyme-linked immunosorbent assay kit, following the manufacturer’s protocol (Human Proprotein Convertase 9/PCSK9 DuoSet ELISA kit; R&D Systems, Minneapolis, MN, USA). The intra- and inter-assay coefficients of variation for PCSK9 levels were 7.01% and 7.02%, respectively (Supplementary Table S1).

### 2.3. Detection of the C378W Mutation Through the Analysis of WGS Data

The WGS data of the TWB cohort were analyzed (secondary analysis) on Illumina (HiSeq 2500/4000), as described by Raczy et al. [27]. Reads were aligned to the hg19 reference genome by using iSAAC (version 01.13.10.21) (Illumina), and variants were identified using iSAAC Variant Caller (2.0.17) [27]. A custom shell script was used to merge 1486 variant call format files into a single data set. A union table of variants was generated for downstream analyses focusing on the *PCSK9* locus to detect the rs776752113 variant.

### 2.4. Polymerase Chain Reaction and Direct DNA Sequencing

Genomic DNA was extracted, and the rs776752113 variant was detected using customized TaqMan single-nucleotide polymorphism (SNP) genotyping assays (Applied Biosystems, Waltham, MA, USA). The variant was subsequently confirmed through Sanger sequencing. Polymerase chain reaction was performed using oligonucleotide primers (forward: 5′-GAGCAGATGCGTACCTGACA-3′; reverse: 5′-CATCAAGCTCCCGATCAAAT-3′) and the KAPA HiFi HotStart PCR kit. Amplified products were sequenced bidirectionally on the ABI PRISM 3730XL DNA Analyzer (Applied Biosystems).

### 2.5. Functional Characterization

The functional implications of the C378W mutation were assessed using predictive tools such as SIFT, PolyPhen-2, SNPs&GO, PANTHER-PSEP, I-Mutant 2.0, MutPred2, PMUT, SNAP-2, Pfam, and Varsome. Gene ontology annotations were incorporated to analyze the mutation’s effects on protein structure, stability, function, and evolutionary conservation. The analysis was performed following established methods [28].

### 2.6. Statistical Analysis

Continuous variables are presented in terms of mean ± standard deviation values. Logarithmic transformation was performed to normalize the variables before regression analyses. Differences in categorical data distribution (sex and smoking status) were examined using a chi-squared test or chi-squared test for trend. Linear regression models were used to identify the associations of the C378W mutation with various lipid traits (TC, LDL-C, HDL-C, non-HDL-C, remnant cholesterol, and PCSK9 levels). To minimize confounding by population stratification, we included the resulting principal components as covariates in the association analyses related to genetic variants. Principal components analysis (PCA) captures major axes of genetic variation that reflect ancestry differences, and adjustment for these components is a standard approach to false positives due to stratification while retaining power to detect true genetic associations [29,30,31]. Thus, the models were adjusted for age, BMI, sex, smoking status, and two principal components (including place of residence and races). Statistical analyses were performed using SPSS (version 22; IBM Corporation, Armonk, NY, USA). Missing values were handled through listwise deletion. The *p* value of less than 0.05 after Bonferroni correction was considered significant.

## 3. Results

### 3.1. Associations of PCSK9 Levels with Clinical Parameters and Lipid Profiles

Table 1 presents the baseline characteristics of the participants, stratified by the availability of WGS data. The average LDL-C and PCSK9 levels in the study cohort (*n* = 5901) were 116.90 ± 31.32 mg/dL and 155.49 ± 47.09 ng/mL, respectively. Participants with a history of hyperlipidemia or a fasting duration of <6 h were further excluded from the analyses of the associations between the lipid profiles, and PCSK9 levels. Baseline characteristics of the remaining 5415 participants are summarized in Supplementary Table S2. The associations of PCSK9 levels with clinical parameters and lipid profiles are presented in Table 2. PCSK9 levels were significantly associated with age, sex, BMI, current smoking, and lipid profiles with higher PCSK9 levels noted in older, obese and smoking participants and in women. Higher lipid levels, such as levels of TC, HDL-C, LDL-C, TG, non-HDL-C and RC, were associated with high PCSK9 levels after adjustment for age, sex, BMI, and current smoking after applying Bonferroni correction.

### 3.2. Results of WGS Data Analysis

The analysis of WGS data from 1486 participants identified 1 carrier of the C378W mutation (Participant 1; Table 3). This participant had a PCSK9 level of 54.02 ng/mL and an LDL-C level of 110 mg/dL.

#### Genotyping of the C378W Mutation in 5901 TWB Participants

**Figure 2:**
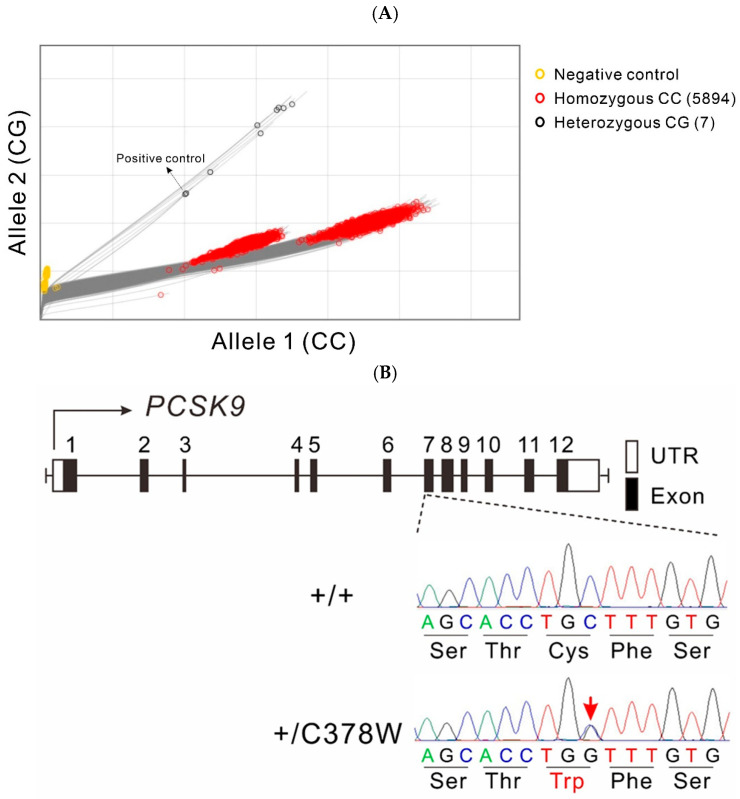
Results of TaqMan SNP genotyping assays and direct DNA sequencing of *PCSK9* C378W mutation. The results of TaqMan SNP genotyping assays in 5901 Taiwan Biobank participants. (**A**): The scatter plot shows allele discrimination for the PCSK9 C378W variant, depicting negative controls (yellow), homozygous CC (red, *n* = 5894), and heterozygous CG carriers (black, *n* = 7). (**B**): The location of the C378W substitution in exon 7 is highlighted. Representative chromatograms show wild-type sequence (+/+) and heterozygous C378W carriers (+/C378W), in which the nucleotide substitution (C→G) results in an amino acid change from cysteine (Cys) to tryptophan (Trp).

TaqMan SNP genotyping assays in 5901 TWB participants detected the heterozygous 378W (rs776752113-G) allele in 7 of them (7/5901 [0.119%]; men: 4; women: 3; Table 3; Figure 2A). All 378W allele carriers had substantially reduced PCSK9 levels (49.66–87.23 ng/mL) compared to the average PCSK9 level in the entire cohort (155.49 ± 47.09 ng/mL). Furthermore, five carriers had LDL-C levels of <85 mg/dL. TaqMan SNP genotyping assays showed complete match with the results of genotyping from 1486 participants having the WGS data. Direct DNA sequencing also confirmed the results of the TaqMan assays for all seven rs776752113-G carriers (Figure 2B and Supplementary Table S3).

### 3.3. Functional Characteristics of the C378W Mutation

Nine computational tools were used to predict the effects of the nonsynonymous C378W mutation on PCSK9 structure, stability, function, and evolutionary conservation (Table 4). Seven of these tools identified the mutation as potentially damaging or pathogenic. Specifically, SIFT (score = 0) and PolyPhen-2 (score = 1) suggested that C378W exerts damaging effects. I-Mutant predicted a reduction in protein stability (Reliability Index = 4). SNPs&GO indicated that the mutation caused disease (Reliability Index = 9). MutPred2 associated the mutation with pathogenic effects (score = 0.723). SNAP-2 predicted a nonneutral effect with 85% accuracy. Varsome yielded a meta score of 6, indicating strong pathogenicity on the basis of evidence from multiple in silico predictors [32]. These findings suggest that the C378W mutation influences biological functions by altering protein stability and causing structural damage, corroborating the results of in vitro and in vivo analyses performed by Meng et al. [26].

### 3.4. Associations of the C378W Mutation with PCSK9 and LDL-C Levels

**Figure 3:**
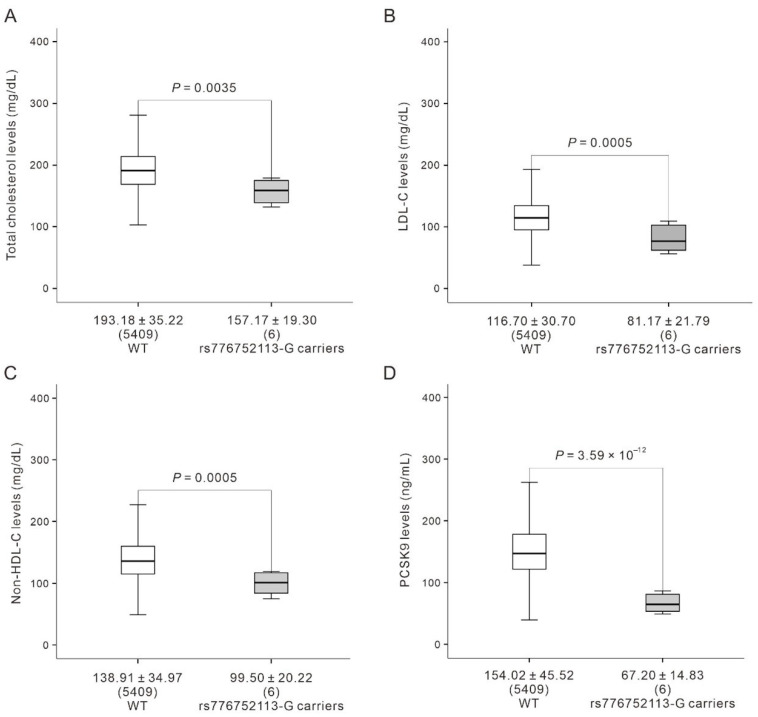
Associations between PCSK9 C378W mutation, PCSK9 levels, and lipid profiles. Levels of (**A**) total cholesterol, (**B**) low-density lipoprotein cholesterol, (**C**) non-high-density lipoprotein cholesterol, and (**D**) PCSK9 in WT participants with rs776752113-CC genotype (*n* = 5409) and rs776752113-G carriers (*n* = 6). Carriers exhibited significantly lower lipid and PCSK9 levels compared to WT.

After the exclusion of a participant with fasting duration of <6 h, the other six individuals carrying the 378W allele had significantly lower levels of TC, LDL-C, non-HDL-C, and PCSK9 than did those carrying the wild-type 378C allele (TC: 157.17 ± 19.30 vs. 193.18 ± 35.22 mg/dL [*p* = 0.0035]; LDL-C: 81.17 ± 21.79 vs. 116.70 ± 30.70 mg/dL [*p* = 0.0005]; non-HDL-C: 99.50 ± 20.22 vs. 138.91 ± 34.97 mg/dL [*p* = 0.0005]; PCSK9: 67.20 ± 14.83 vs. 154.02 ± 45.52 ng/mL [*p* = 3.59 × 10^−12^]). These differences correspond to reductions of 56.4%, 18.6%, 30.5%, and 28.4% in the levels of PCSK9, TC, LDL-C, and non-HDL-C in participants carrying the 378W allele (Figure 3A–D). Mediation analyses were performed to determine whether PCSK9 levels mediated the associations of the C378W mutation with lipid profiles. After further adjustment for PCSK9 levels, the association between the C378W mutation and LDL-C and non-HDL-C levels remained significant, suggesting that the effects of the mutation on LDL-C and non-HDL-C levels were independent to PCSK9 levels (Table 5).

## 4. Discussion

This study provides compelling evidence that the *PCSK9* C378W mutation is a rare, Asian-specific variant associated with substantially reduced plasma PCSK9 levels and improved lipid profiles (corresponding to marked reductions in LDL-C, non-HDL-C, and TC levels). Our findings indicate the cardioprotective role of the C378W mutation and support its classification as a loss-of-function mutation. Despite its rarity, the mutation is biologically important, as evidenced by the significant reduction in the levels of PCSK9 (−56.4%) and LDL-C (−30.5%) in carriers. This magnitude of the effect aligns with those reported by studies identifying loss-of-function *PCSK9* variants as key contributors to reduced cardiovascular risks [33,34,35]. Notably, the significant reductions we observed in the levels of non-HDL-C and RC reinforce the potential cardiovascular benefits of the C378W mutation.

### 4.1. Minor Allele Frequency of the C378W Mutation

Our analysis revealed that the C378W mutation with the rs776752113-G allele was a rare mutation with an allele frequency of 0.119% in the study cohort. According to PubMed, the frequency of the rare rs776752113-A allele among 32,642 European individuals is 0.006%. By contrast, the frequencies of the rare rs776752113-G allele in two Japanese populations of 28,258 and 16,760 individuals are 0.004% and 0.006%, respectively. None of the other populations studied had the mutation at the C378 position of *PCSK9*. Meng et al. [26] detected the C378W mutation in a Uyghur family. A subsequent population genetic analysis involving 2483 additional Uyghur individuals, 2784 Han individuals, and 223 Kazakh individuals revealed the heterozygous C378W mutation in two (2/2784 [0.072%]), Han Chinese individuals but not in the other two populations. In the present study, we detected the 378W (G) allele in 7 of 5901 participants (0.119%); direct DNA sequencing confirmed that all carriers had the C-to-G mutation. These findings suggest a higher prevalence of the C378W mutation in the Han Chinese population relative to other ethnic groups, underscoring the presence of ethnic-specific genetic heterogeneity. The identification of rare mutations such as C378W in the TWB cohort highlights the importance of large-scale genomic studies in uncovering clinically significant genetic variants. Despite its low prevalence, the C378W mutation provides a natural model for studying the effects of PCSK9 inhibition, offering potential translational insights for personalized medicine.

### 4.2. Association Between the C378W Mutation and PCSK9 Levels

In our study, all seven carriers of the rs776752113-G allele had significantly lower PCSK9 levels than noncarriers. The average level of PCSK9 in the carriers was only 43.6% of that observed in noncarriers (67.20 vs. 154.0 ng/mL). These results are consistent with those of an in vitro study conducted using HEK293T cells and primary hepatocytes, where the mature PCSK9 protein with the C378W mutation, introduced through transfection, formed with an impaired endoplasmic reticulum exit, which prevented its secretion into the medium. Additionally, an animal study involving adeno-associated virus-mediated expression of the C378W form of human PCSK9 in *pcsk9* knock-out mice revealed marked reductions in the serum levels of PCSK9 compared with the levels in *pcsk9* knock-out mice transfected with wild-type human PCSK9 [26]. These findings suggest that the C378W mutation strongly reduces PCSK9 levels in both humans and experimental animals.

### 4.3. Association Between the C378W Mutation and LDL-C Levels

Elevated LDL-C levels are associated with increased risks of atherosclerotic cardiovascular disease, cardiovascular mortality, and all-cause mortality [36,37]. Reducing LDL-C levels considerably reduces morbidity and mortality in patients with atherosclerotic cardiovascular disease [38,39]. A reduction of 0.35 mmol/L (13.5 mg/dL) in LDL-C levels through genetic variations can reduce the lifetime risk of atherosclerotic cardiovascular disease by 20% [40]. In our study, carriers of the rs776752113-G allele exhibited a reduction of 35.2 mg/dL (or 30.4%) in LDL-C levels compared with the levels in noncarriers (81.17 vs. 116.70 mg/dL). This reduction is similar to that observed by Meng et al. in carriers of the rs776752113-G allele [26]. Therefore, carrying the rs776752113-G allele may reduce the lifetime risk of atherosclerotic cardiovascular disease by 52%, calculated on the basis of LDL-C levels. Notably, two carriers (participants 1 and 5; Table 3) identified in the analysis of WGS data exhibited substantially low PCSK9 levels, but only mild reductions in LDL-C levels. This discrepancy underscores the complexity of lipid metabolism and the influence of other genetic or environmental factors on LDL-C levels. Further large-scale studies are warranted to clarify these interactions.

### 4.4. Mediation Effect of PCSK9 Levels on the Association Between C378W Mutation and LDL-C Level

The C378W mutation prevents PCSK9 from entering the secretory pathway, thereby reducing its circulating level and inhibiting LDL receptor degradation [26]. To determine whether the effect of the C378W mutation on LDL-C level is mediated by level of PCSK9, we investigated the associations between the mutation, PCSK9 level, and LDL-C level. Even after adjustments for PCSK9 level, the association between the C378W mutation and LDL-C level remained significant. These findings imply that the reduction in LDL-C level in C378W mutation carriers is only partially driven by reduced PCSK9 level. The LDL-C level is influenced by genetic variants across multiple lipid metabolism–related pathway genes, while environmental and metabolic factors may also contribute, collectively accounting for the heterogeneity in LDL-C reduction observed among carriers [41,42,43]. Because both PCSK9 and LDL-C levels independently influence long-term clinical outcomes [44,45], carriers of the rs776752113-G allele may receive additional cardiovascular benefits from the combined effects of reduced PCSK9 and LDL-C levels. Recent clinical outcome trials have demonstrated that the PCSK9 inhibitor therapy significantly reduce the risk of major adverse cardiovascular events in patients with atherosclerotic cardiovascular disease [46,47]. Given the partial mediation observed, future studies may benefit from systems-level modeling approaches to simulate the nonlinear dynamics between PCSK9, LDLR, and lipid traits, as demonstrated in chemo-immunotherapy contexts [48].

### 4.5. Limitations

This study has several limitations. First, its cross-sectional design precluded the assessment of long-term cardiovascular outcomes in mutation carriers. Second, because of genetic heterogeneity across ethnicities, our findings may not be applicable to other ethnic populations. Third, although bioinformatic tools provide valuable predictions, in vitro or in vivo functional validation is necessary to confirm the mechanistic effects of the mutation. Meng et al. [26] reported that the C378W mutation attenuated the binding of PCSK9 to SURF4 (surfeit 4) and its subsequent egress from the endoplasmic reticulum, which prevented the secretion of mature PCSK9 into the medium and the PCSK9-induced degradation of LDL receptor. Additional functional assays, particularly evaluations of PCSK9 secretion in hepatocyte models, are warranted to substantiate and reinforce the conclusions. Fourth, a key limitation of this study is the very small number of C378W carriers identified (*n* = 7), which restricts statistical power and raises the possibility of both type I and type II errors, a common challenge in rare variant studies. Although the effect sizes observed for LDL-C and PCSK9 were large and biologically consistent, replication in larger cohorts or through meta-analyses will be essential to validate these findings and to fully establish the clinical relevance of this mutation. Finally, this study focused on only a single population; replication in other populations is warranted.

## 5. Conclusions

The present study suggests that the *PCSK9* C378W mutation is a rare but important loss-of-function mutation independently associated with reduced PCSK9 and LDL-C levels in the Taiwanese population. The combined effects of this mutation on both PCSK9 and LDL-C levels may help to predict the risk of cardiometabolic diseases. Future studies should elucidate the roles of functional *PCSK9* variants in shaping disease outcomes.

## Figures and Tables

**Table 1:** Baseline characteristics of 5901 Taiwan Biobank participants.

	Total Study Participants	Participants with WGS	Participants Without WGS
Number	5901	1486	4415
Age (years)	48.82 ± 11.08	49.46 ± 11.27	48.60 ± 11.01
Sex (M/F)	2703/3198 (=5901)	745/741 (=1486)	1958/2457 (=4415)
BMI (kg/m^2^)	24.24 ± 3.64	24.36 ± 3.70	24.19 ± 3.62
Current smoking (Yes/No)	632/5269 (10.71%)	150/1336 (10.09%)	482/3933 (10.92%)
TC (mg/dL)	193.75 ± 35.65	194.46 ± 35.21	193.53 ± 35.79
LDL-C (mg/dL)	116.90 ± 31.32	118.25 ± 31.64	116.45 ± 31.19
HDL-C (mg/dL)	53.96 ± 13.22	53.81 ± 13.91	54.01 ± 12.98
TG (mg/dL)	116.62 ± 98.12	113.93 ± 91.27	117.53 ± 100.32
RC (mg/dL)	22.90 ± 17.43	23.39 ± 15.63	23.07 ± 17.99
Non-HDL-C (mg/dL)	139.80 ± 35.42	140.65 ± 34.48	139.52 ± 35.73
PCSK9 level (ng/mL)	155.49 ± 47.09	159.63 ± 48.21	154.06 ± 46.59

WGS, whole genomic sequencing; BMI, body mass index; TC, total cholesterol; LDL-C, low-density lipoprotein cholesterol; HDL-C, high-density lipoprotein cholesterol; TG, triglyceride; RC, remnant cholesterol.

**Table 2:** Associations of PCSK9 levels with clinical parameters and lipid profiles in 5415 Taiwan Biobank participants.

Clinical and Laboratory Parameters	Beta	SE	*p*	Adjusted *p* *
Anthropology				
Age (years) ^a^	0.0021	0.0002	1.16 × 10^−39^	1.16 × 10^−38^
BMI (kg/m^2^) ^b^	0.0034	0.0005	1.61 × 10^−12^	1.61 × 10^−11^
Sex (Female/Male) ^c^	1.9735	0.2382	1.19 × 10^−16^	1.19 × 10^−15^
Current smoking (Yes/No) ^d^	1.1768	0.3737	0.0016	0.016
Lipid profiles ^e^				
TC (mg/dL)	0.2611	0.0225	1.07 × 10^−30^	1.07 × 10^−29^
HDL-C (mg/dL)	0.0332	0.0190	0.0038	0.038
LDL-C (mg/dL)	0.1209	0.0150	1.10 × 10^−15^	1.10 × 10^−14^
TG (mg/dL)	0.1236	0.0081	1.83 × 10^−51^	1.83 × 10^−50^
Non-HDL-C (mg/dL)	0.1862	0.0166	7.61 × 10^−29^	7.61 × 10^−28^
RC (mg/dL)	0.0377	0.0059	1.29 × 10^−10^	1.29 × 10^−9^

*p*: ^a^ Adjusted for sex, BMI, and smoking status; ^b^ adjusted for age, sex, and smoking status; ^c^ adjusted for age, BMI, and smoking status; ^d^ adjusted for age, sex, and BMI; ^e^ adjusted for age, sex, BMI, and smoking status. * Adjusted *p* value: Adjusted with Bonferroni correction, *n* = 10. BMI, body mass index; TC, total cholesterol; LDL-C, low-density lipoprotein cholesterol; HDL-C, high-density lipoprotein cholesterol; TG, triglyceride; RC, remnant cholesterol.

**Table 3:** Lipid profiles and PCSK9 levels in Taiwan Biobank participants carrying *PCSK9* C378W mutation.

Participants	Age (Years)	Sex	BMI (kg/m^2^)	History of Hyperlipidemia	Smoking Status	TC (mg/dL)	LDL-C (mg/dL)	HDL-C (mg/dL)	TG (mg/dL)	PCSK9 (ng/mL)
1	34	Male	23.26	No	No	175	114	56	46	54.02
2	47	Female	26.94	No	No	166	94	49	163	87.23
3	49	Female	22.66	No	No	152	71	67	74	65.34
4	57	Male	23.54	No	No	132	48	48	136	81.70
5	63	Female	23.44	No	No	179	107	62	69	65.27
6	68	Male	24.80	No	No	139	64	64	62	49.66
7	42	Male	21.55	No	No	141	81	50	73	75.89

TC, total cholesterol; LDL-C, low-density lipoprotein cholesterol; HDL-C, high-density lipoprotein cholesterol; TG, triglyceride.

**Table 4:** Functional characteristics of *PCSK9* C378W (rs776752113) mutation.

Tools	Information Used for Prediction	Score or Reliability Index Value	Probability/Prediction	Threshold	Remark
SIFT	Conserved region	0	Damaging	Deleterious if ≤0.05	
Polyphen-2 (HumDiv)	Structural homology	1	Damaging		Sensitivity: 0.00; specificity: 1.00
PolyPhen-2 (Humvar)		0.986	Damaging		Sensitivity: 0.54; specificity: 0.94
SNPs&GO	Functional information categorized by gene ontology	9	Disease		
PANTHER-PSEP	Evolutional preservation	1 (preservation time)	Probably benign	>450 million years ago	0.02 (probability of deleterious effects)
I-Mutant 2.0	Changes in structural stability	4 (Reliability Index)	Reduced stability		
MutPred2	Sequence information for labeling mutations and neural networks for processing the information	0.723	Probability (predicted conservation scores)	Molecular mechanisms with a *p* value of ≤0.05	
SNAP-2	Networks for assessing the effects of single-amino-acid substitutions	78	Effect (predicted effect)		85% (expected accuracy)
PMUT	Machine learning model and software package integrating genetic and molecular data	0.40 (86%)	Neutral effect		
Varsome	Information from the Global Genomics Community of >500,000 health-care professionals and researchers; the tool features a massive knowledge base comprising 140+ data resources and a powerful variant search engine	Point score: 6	Pathogenic strong	Engines are assigned a prediction score on the basis of the strength of the calibrated prediction *	The predictors determine pathogenicity on the basis of combined evidence from multiple other in silico predictors

* Prediction score: 1 point: supporting; 2 points: moderate; 4 points: strong; 8 points: very strong.

**Table 5:** Associations between rs776752113 variant, PCSK9 levels, and lipid profiles.

	CC (5409)	CG (6)	β	SE	*P*_1_	Adjusted *P*_1_ *	β	SE	*P*_2_	Adjusted *P*_2_ *
TC (mg/dL)	193.18 ± 35.22	157.17 ± 19.30	−0.0907	0.0311	0.0035	0.0245	−0.058	0.0309	0.0609	0.4263
TG (mg/dL)	112.72 ± 94.33	91.67 ± 46.57	−0.0644	0.0859	0.4532	3.1724	0.0618	0.0844	0.4638	3.2466
HDL-C (mg/dL)	54.26 ± 13.24	57.67 ± 7.97	0.0355	0.0373	0.3419	2.3933	0.0414	0.0375	0.2701	1.8907
LDL-C (mg/dL)	116.70 ± 30.70	81.17 ± 21.79	−0.165	0.0474	0.0005	0.0035	−0.1396	0.0475	0.0033	0.0231
Non-HDL-C (mg/dL)	138.91 ± 34.97	99.50 ± 20.22	−0.1472	0.0423	0.0005	0.0035	−0.1038	0.042	0.0135	0.0945
RC (mg/dL)	22.22 ± 17.09	18.33 ± 9.31	−0.0639	0.0842	0.4477	3.1339	0.061	0.0826	0.4606	3.2242
PCSK9 level (ng/mL)	154.02 ± 45.52	67.20 ± 14.83	−0.3527	0.0506	3.59 × 10^−12^	2.51 × 10^−11^	−0.3392	0.0505	2.12 × 10^−11^	1.48 × 10^−10^

*P*_1_: Adjusted for age, body mass index, sex, smoking status, and two principal components. *P*_2_: Additionally adjusted for PCSK9 levels when analyzing lipid profiles, and for LDL-C levels when analyzing PCSK9 levels. * Adjusted *P*_1_ and *P*_2_: Adjusted with Bonferroni correction, *n* = 7. TC, total cholesterol; LDL-C, low-density lipoprotein cholesterol; HDL-C, high-density lipoprotein cholesterol; TG, triglyceride; RC, remnant cholesterol. Note: Participants were excluded from the analysis if they had a history of hyperlipidemia or a fasting duration of <6 h.

## Data Availability

The original contributions presented in this study are included in the article/Supplementary Material. Further inquiries can be directed to the corresponding author.

## Supplementary Materials

The following supporting information can be downloaded at: https://www.mdpi.com/article/10.3390/genes16091113/s1, Table S1: Intra- and inter-assay plasma PCSK9 level variability; Table S2: Baseline characteristics of the study cohort after the exclusion of individuals with a history of hyper-lipidemia or a fasting duration of <6 h; Table S3: Direct DNA sequencing result of 3 participants for normal (N1–N3) and 7 participants for PCSK9 C378W mutation (1–7).

## Abbreviations

The following abbreviations are used in this manuscript:

PCSK9	Proprotein convertase subtilisin/kexin type 9
LDL	low-density lipoprotein
LDL-C	low-density lipoprotein cholesterol
TWB	Taiwan Biobank
WGS	whole-genome sequencing
BMI	body mass index
TC	total cholesterol
HDL-C	high-density lipoprotein cholesterol
TG	triglycerides
SNP	single-nucleotide polymorphism
RC	remnant cholesterol
