# Supplementary material for: Effects of the PCSK9 C378W Mutation on PCSK9 Levels and Lipid Profiles in Taiwanese Individuals: A Loss-of-Function Mutation with Potential Cardiovascular Benefits
Source: Genes (Basel). 2025 Sep 19;16(9):1113. doi: 10.3390/genes16091113 (PMC12470178; doi:10.3390/genes16091113)
Supplement: Supplementary file 1 [file genes-16-01113-s001.zip › genes-3855460-supplementary.pdf]

Supplementary Material

Table S1. Intra- and inter-assay plasma PCSK9 level variability.

Biomarker	Source	Intra-assay	Inter-assay
PCSK9	Plasma	CV = 7.01%	CV = 7.02%

CV, coefficient of variation.

Table S2. Baseline characteristics of the study cohort after the exclusion of individuals with a history of hyperlipidemia or a fasting duration of <6 h.

	Total study participants	Participants with WGS	Participants without WGS
Number	5415	1366	4049
Age (years)	48.35 ± 11.04	49.08 ± 11.26	48.10 ± 10.96
Sex (M/F)	2445/2970	671/695	1774/2275
BMI (kg/m^2)	24.14 ± 3.60	24.25 ± 3.63	24.11 ± 3.58
Current smoking (Yes/No)	577/4838 (10.66%)	141/1225 (10.32%)	436/3613 (10.77%)
TC (mg/dL)	193.14 ± 35.22	193.61 ± 34.45	193.00 ± 35.47
LDL-C (mg/dL)	116.66 ± 30.72	117.82 ± 30.50	116.28 ± 30.78
HDL-C (mg/dL)	54.27 ± 13.24	54.11 ± 13.86	54.32 ± 13.02
TG (mg/dL)	112.69 ± 94.29	109.52 ± 83.11	113.77 ± 97.77
RC (mg/dL)	22.21 ± 17.08	21.67 ± 14.88	22.39 ± 17.76
Non-HDL-C (mg/dL)	138.88 ± 34.97	139.49 ± 33.51	138.68 ± 33.45
PCSK9 level (ng/mL)	153.92 ± 45.59	157.57 ± 46.07	152.65 ± 45.32

WGS, whole genomic sequencing; BMI, body mass index; TC, total cholesterol; LDL-C, low-density lipoprotein cholesterol; HDL-C, high-density lipoprotein cholesterol; TG, triglyceride; RC, remnant cholesterol.

Table S3. Direct DNA sequencing result of three participants for normal (N1-N3) and seven participants for *PCSK9* C378W mutation (1–7).

**Samples**	**Participants**	**Results**	**Genotypes**
+/+	N1	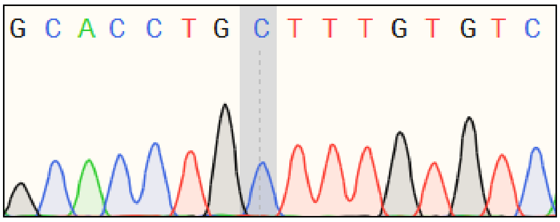	C/C
+/+	N2	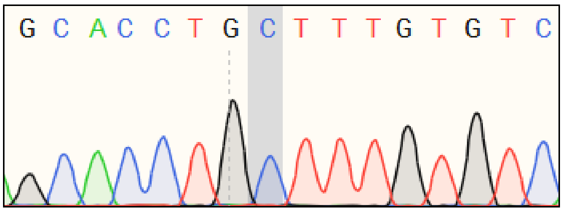	C/C
+/+	N3	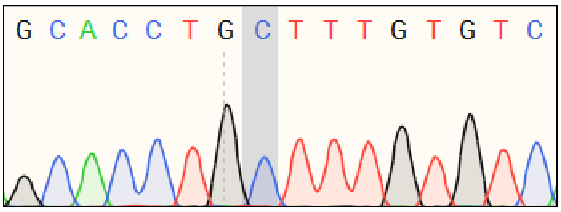	C/C
+/C378W	1	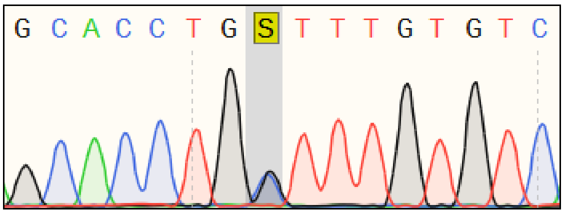	C/G

+/C378W 2 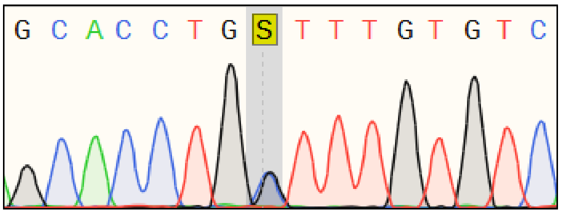 C/G

+/C378W 3 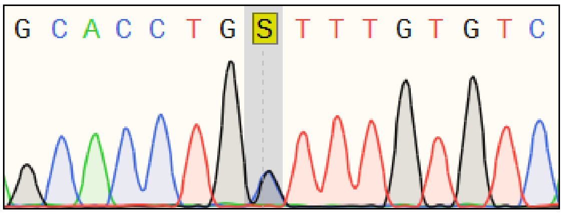 C/G

+/C378W 4 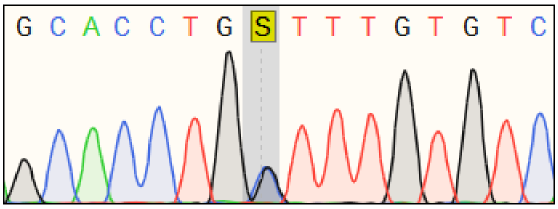 C/G

+/C378W 5 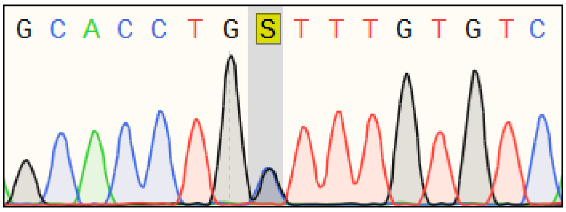 C/G

+/C378W 6 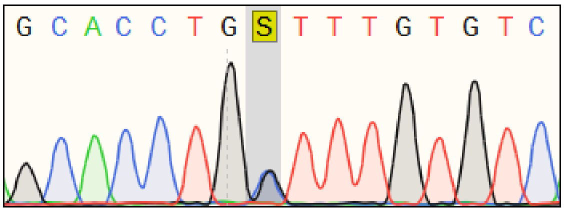 C/G

+/C378W	7	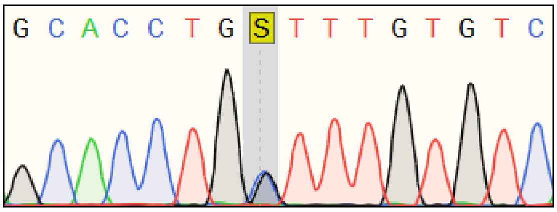	C/G
